# Mendelian randomization with Egger pleiotropy correction and weakly informative Bayesian priors

**DOI:** 10.1093/ije/dyx254

**Published:** 2017-12-15

**Authors:** A F Schmidt, F Dudbridge

**Affiliations:** 1 Groningen Research Institute of Pharmacy, University of Groningen, Groningen, The Netherlands; 2 Institute of Cardiovascular Science, University College London, London, UK; 3 Department of Cardiology, University Medical Center Utrecht, Utrecht, The Netherlands; 4 Department of Health Sciences, University of Leicester, Leicester, UK; 5 Department of Non-Communicable Disease Epidemiology, London School of Hygiene and Tropical Medicine, London, UK

**Keywords:** Epidemiology methods, Bayesian analysis, Mendelian randomization, instrumental variables, pleiotropy

## Abstract

**Background:**

The MR-Egger (MRE) estimator has been proposed to correct for directional pleiotropic effects of genetic instruments in an instrumental variable (IV) analysis. The power of this method is considerably lower than that of conventional estimators, limiting its applicability. Here we propose a novel Bayesian implementation of the MR-Egger estimator (BMRE) and explore the utility of applying weakly informative priors on the intercept term (the pleiotropy estimate) to increase power of the IV (slope) estimate.

**Methods:**

This was a simulation study to compare the performance of different IV estimators. Scenarios differed in the presence of a causal effect, the presence of pleiotropy, the proportion of pleiotropic instruments and degree of ‘Instrument Strength Independent of Direct Effect’ (InSIDE) assumption violation. Based on empirical plasma urate data, we present an approach to elucidate a prior distribution for the amount of pleiotropy.

**Results:**

A weakly informative prior on the intercept term increased power of the slope estimate while maintaining type 1 error rates close to the nominal value of 0.05. Under the InSIDE assumption, performance was unaffected by the presence or absence of pleiotropy. Violation of the InSIDE assumption biased all estimators, affecting the BMRE more than the MRE method.

**Conclusions:**

Depending on the prior distribution, the BMRE estimator has more power at the cost of an increased susceptibility to InSIDE assumption violations. As such the BMRE method is a compromise between the MRE and conventional IV estimators, and may be an especially useful approach to account for observed pleiotropy.

**Key Messages**

- Absence of pleiotropy is an essential assumption for instrumental variable analyses using genetic instruments, known as Mendelian randomization. The MR-Egger method corrects for the presence of pleiotropy by introducing a nuisance parameter which captures directional pleiotropy. However, including this nuisance parameter greatly reduces power to detect a causal effect as compared to the traditional inverse variance weighted (IVW) estimator.
- In this paper we propose a novel Bayesian implementation of the MR-Egger, ‘BMR-Egger’, which increases the power of the causal estimate by introducing a weakly informative prior on the nuisance parameter. Our motivation is that the BMR-Egger can be seen as a compromise between two extreme prior distributions. Specifically, the IVW method corresponds to applying an optimistic informative prior on the intercept with a mean and variance of zero, whereas MR-Egger corresponds to a pessimistic non-informative prior with an infinite variance.
- When the ‘Instrument Strength Independent of Direct Effect’ (InSIDE) assumption holds, the BMR-Egger has increased power with acceptable type 1 error rates as compared to the MR-Egger. If the InSIDE assumption is violated, all estimators are biased and show inappropriately high rejection rates. In this case, adding prior beliefs increases bias and rejection rates of the BMR-Egger towards that of the IVW estimator.

## Introduction

Instrumental variable analyses using genetic instruments, often termed Mendelian randomization (MR) analyses, use genetic exposures as instruments to determine the causal association between an intermediate phenotype, often a biomarker, and a particular outcome such as disease. The estimate of such an MR analysis reflects an unbiased causal estimate of the phenotype effect on the outcome, if (among others) the following assumptions are met.

- The instruments are associated with the phenotype.
- The instruments are independent of observed and unobserved confounders of the phenotype-outcome association.
- Conditional on the phenotype and confounders, the instruments are independent of the outcome (i.e. the exclusion restriction assumption).

Given that biomarkers are the (indirect) products of multiple genes, it is often possible to select a set of genetic instruments that meet assumption (i). Furthermore, because genes are randomly allocated at conception,^1^ assumption (ii) is often plausible as well. Assumption (iii) states that the genes can only be related to disease due to their effects on the phenotype (i.e. no pleiotropy other than that mediated by the phenotype). Whether this assumption generally holds has been contested.^2^ For example, if one is interested in estimating the causal relation between high-density lipoprotein cholesterol (HDL-C) and coronary heart disease (CHD), it is often difficult to find genes that affect HDL-C but not low-density lipoprotein cholesterol (LDL-C) or triglycerides.^3^^,^^4^ Such a situation may indicate violation of assumption (ii) (when LDL-C is viewed as a confounder of HDL-C and CHD), of assumption (iii) (when a gene effects both pathways independently) or of both assumptions. In practice, such distinctions are difficult to make and hence robust IV methods are preferred.

Recently Bowden *et al.*^5^ proposed a novel method related to the Egger test,^6^ ‘Mendelian randomization Egger’ (MR-Egger/MRE), which corrects for potential violations of assumption (iii) by quantifying the amount of directional pleiotropy. This MR-Egger method assumes that the ‘Instrument Strength is Independent of the Direct Effect’ (i.e. the InSIDE assumption), which means that across single nucleotide polymorphisms (SNPs), pleiotropic effects are independent of phenotypic effects. The MR-Egger method corrects for pleiotropy by introducing a nuisance parameter which quantifies the average amount of directional pleiotropy. However, including this nuisance parameter greatly reduces precision and power to detect a causal effect. Despite this reduced power, the MRE method has been frequently used in empirical settings.^4^^,^^7–9^

In this paper, we propose a novel Bayesian implementation of the MR-Egger method, ‘BMR-Egger’, which increases power of the causal estimate by introducing a (weakly) informative prior on the nuisance parameter, which is the intercept in a linear regression. From a Bayesian perspective, the standard inverse variance weighted (IVW) estimator and the MRE estimator can be unified by noticing that the IVW method corresponds to putting an optimistic informative prior on the intercept with mean and variance of zero; conversely, the MRE approach can be seen as a pessimistic non-informative prior with infinite variance. Whereas pessimistic and optimistic priors are often used, for example in randomized controlled trials (RCTs) in rare diseases,^10^ in genetics considerable data may be available on the magnitude of pleiotropy and consequently less extreme, more believable priors may be usefully employed.

One reasonable approach may be a prior belief that extreme departures from balanced pleiotropy are unlikely, as strong pleiotropic effects of genetic variants may have been previously identified. This is similarly optimistic to the IVW method; however, instead of (unrealistically) assuming a zero prior variance, we suggest use of weakly informative priors to allow for a degree of pleiotropy. Alternatively, as we will discuss using an empirical example of urate and coronary heart disease (CHD),^7^ often considerable (aggregated) data will be available on potential pleiotropic pathways, which can be used to further elucidate a prior distribution to fit the specific data at hand. We note that defining what constitutes a pleiotropic pathway is difficult and will depend on subjective criteria such as statistical significance and the availability of relevant datasets (such as MR-base^11^).

In the following, we introduce notations, and the outcome model, followed by a review of the MR estimators and the proposed novel Bayesian MR estimator. Subsequently we evaluate the discussed methods in a simulation study, and the empirical example noted above.

## Methods

### Notation, and outcome model

Let us assume there are data available from j=1,…,J independent single-nucleotide polymorphisms (SNPs) G (an i=1,…,n subject by J matrix), with $\alpha_{j}$ representing the (marginal) effect of SNP j on a biomarker X, $\beta_{j}$ the (marginal) SNP effect on an outcome Y, and variance of their estimators $\sigma_{\alpha_{j}}^{2}$ and $\sigma_{\beta_{j}}^{2}$. We note that $\alpha_{j}$ and $\beta_{j}$ may be estimated from the same data (one-sample MR study) or in separate data (two-sample MR study); we focus on the latter.^12^ Based on these data we are interested in estimating the causal effect of X on Y, assuming Y is generated by the linear model $Y_{i}=\overset{J}{\underset{j}{\sum }}\varphi_{j}G_{ij}+\theta X_{i}+{ɛ}_{y}$, with θ a scalar, and Y,G and X as defined above. When assumptions (ii–iii) hold, $\varphi_{j}=0$ and $Y_{i}=\theta X_{i}+{ɛ}_{y}$. Note that the absence of an intercept term in the above equations should be interpreted as meaning the intercept (arbitrarily) equals zero, and should not be misinterpreted as an absence of pleiotropy which is represented by $\varphi_{j}.$

### MR estimators

When there are multiple instruments available, the causal (IV) effect of X on Y can be estimated using a weighted ordinary least squares (OLS) regression of $\beta_{j}$ on $\alpha_{j}$ while supressing the intercept. Given that $\beta_{j}$ and $\alpha_{j}$ are unknown, they are estimated from the data, with the estimates collected in the following matrices:

A = [a^1⋮a^J],B = [β^1⋮β^J],Ωjk = σ^βjσ^βkpjk,

where Ω is the sample variance-covariance matrix for *B*, with $p_{j=k}=1$ and, assuming that SNPs are independent, $p_{j\neq k}=0$. In the case of correlated SNPs, $p_{j\neq k\;}$can be estimated based on the pairwise between SNP correlations^13^ and the regression fitted by generalized least squares. The following regression is weighted by the precision of the SNP effect estimates, giving the IVW point estimate and standard error estimates (assuming no pleiotropy, or balanced positive and negative pleiotropic effects under the InSIDE assumption)^14^ as:

(1) $${\hat{\theta }}_{IVW}\;=\;{\left(A^{t}{\text{Ω}}^{-1}A\right)}^{-1}A^{t}{\text{Ω}}^{-1}B,$$

with the weighted residuals:

$${\hat{ɛ}}_{j}\;=\;diag{\left({\text{Ω}}^{-1}\right)}^{\frac{1}{2}}\left(B-{\hat{\theta }}_{IVW}\text{A}\right),$$

where diag(·) indicates the diagonal elements. The variance of the error term is then:

$${\hat{\sigma }}_{ɛ}^{2}\;=\;\frac{1}{J-k}+\overset{J}{\underset{j\;=\;1}{\sum }}{\hat{ɛ}}_{j}^{2},$$

where k equals the number of parameters (k=1 in this specific case). Finally, the standard error of the slope is:

(2) $${\hat{\sigma }}_{{\text{θ}}_{IVW}}\;=\;diag{\left({\hat{\sigma }}_{ɛ}^{2}{\left(A^{t}{\text{Ω}}^{-1}A\right)}^{-1}\right)}^{\frac{1}{2}}.$$

Here, and in the following derivations, the sigma term ${\hat{\sigma }}_{ɛ}^{2}$ will only be included if it is larger than 1, resulting in standard errors following a multiplicative random effects model.^15^

The MR-Egger method corrects for (unmeasured) directional pleiotropy by introducing an intercept term which captures the expected effect of an instrument on outcome when it has no effect on the biomarker, and is hence a measure of the average amount of pleiotropy. To implement the MR-Egger we first recode the data as follows:

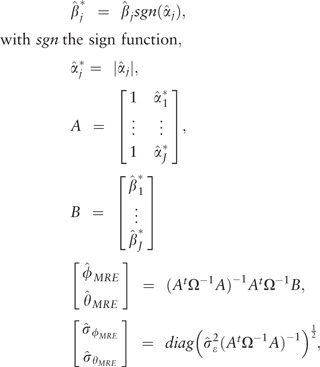

with ${\hat{\sigma }}_{ɛ}^{2}$ derived as before, ${\hat{\theta }}_{MRE}$ the slope estimate, and ${\hat{\varphi }}_{MRE}$ the intercept estimate.

Next, we describe our proposed Bayesian MR-Egger method [BMRE] using a bivariate normal likelihood and the conjugate prior distribution with hyperparameters for the prior mean and variance of the intercept and slope:

μ0 = [μφμθ]Σ0 = [σμφ2 00σμθ2].

Then the posterior distribution is bivariate normal with mean $\mu_{N}$ and variance-covariance matrix ${\text{Σ}}_{N}$:

λ0 = Σ0−1,μN = (AtΩ−1A+λ0)−1(λ0μ0t+AtΩ−1B),ΣN = σ^ɛ2(AtΩ−1A+λ0)−1,

with ${\hat{\sigma }}_{ɛ}^{2}$ derived as before.

To explore the effect of including prior information using weakly informative priors, we performed the simulation study described below. Specifically, we were interested in exploring the advantage of specifying a prior on the intercept ${\hat{\varphi }}_{BMRE}$ to increase precision of the posterior ${\hat{\theta }}_{BMRE}$ and on the robustness of prior misspecification. In our empirical example, we illustrate how to use empirical data on observed pleiotropy signals to elucidate reasonable priors, decreasing the likelihood of prior misspecification.

Our results will also discuss a further method to allow for pleiotropy, the weighted median (WM) estimator.^16^ This estimator assumes that at least 50% of the weights, $w_{j}=\frac{{\hat{\alpha }}_{j}^{2}}{{\hat{\sigma }}_{\beta_{j}}^{2}}$, come from valid instruments. If this assumption is true, a consistent estimate of causal effect is the 50th percentile of the empirical distribution function of SNP-specific IV estimates $\frac{{\hat{\alpha }}_{j}}{{\hat{\beta }}_{j}}$, with the percentile distribution based on $\frac{s_{j}-\frac{w_{j}}{2}}{S}$; where $s_{j}=\overset{j}{\underset{k\;=\;1}{\sum }}w_{k}$, the cumulative sum up to the jth SNP, and $S\;=\;\overset{J}{\underset{k\;=\;1}{\sum }}w_{k}$. 

### Data-generating process

Similar to the original publication by Bowden *et al.*,^5^ data for i = 1, …, n subjects were simulated, with n = 1000 and J = 20 SNPs. $G_{ij}$ were sampled from a trinomial distribution with minor allele frequency $p_{j}=0.30\;$under Hardy–Weinberg equilibrium. An unmeasured confounder was generated based on $U_{i}\;=\;\overset{J}{\underset{j}{\sum }}\omega_{1j}G_{ij}+\varepsilon_{u};\;\varepsilon_{u}∼N\left(0,2\right)$ and a biomarker $X_{i}=\overset{J}{\underset{j}{\sum }}\alpha_{j}G_{ij}+\omega_{2}U_{i}+\varepsilon_{x};\;\varepsilon_{x}∼N\left(0,2\right).$ Finally, the outcome was generated following $Y_{i}=\;\overset{J}{\underset{j}{\sum }}\varphi_{j}G_{ij}+\theta X_{i}+\omega_{3}U_{i}+\varepsilon_{y};\;\varepsilon_{y}∼N\left(0,2\right).$ Based on the two-sample MR principle,^12^ this algorithm was run twice (with the same parameters) to generate two independent datasets, the first used to derive the genetic effects on the biomarker by fitting the linear model $X_{i}=\alpha_{j}G_{ij}+\varepsilon_{x}$, and the second to estimate the genetic effects on the outcome from the linear model $Y_{i}\;=\;\beta_{j}G_{ij}+\varepsilon_{y}$.

### Simulation scenarios

The above defined MR estimators were evaluated in five scenarios (Table 1). In scenario I there was no pleiotropy, hence $\varphi_{j}\;=\;0$, and the confounder was independent of the SNPs, $\omega_{1j}\;=\;0$. In scenario II pleiotropy was generated based on $\varphi_{j}∼U\left(0.00,\;0.20\right)$, and in scenario III the InSIDE assumption was violated by setting $\omega_{1j}∼U\left(L,U\right),L=0,U=0.50.$ In scenario IV the InSIDE assumption was met, $\omega_{1j}=0$, and pleiotropy was generated based on $\varphi_{j}∼U(0.00,0.50)$ with probability q={0.1, 0.2,0.3, 0.4} and 0 otherwise, resulting in (on average) qJ SNPs violating assumption (iii). In this scenario the average pleiotropy depends on q and ranged between $E\left(\varphi_{j}\right)q\;=\;\{0.025,\;\ldots ,\;0.100\}$. Subsequently, in scenario V q = 0.4 with $\varphi_{j}$ and $\omega_{1j}$ generated based on q as in scenario IV. Different types and severities of InSIDE assumption violations were generated by first setting L = 0 and B = {0.10, 0.30, 0.60, 1.00}, and subsequently setting L = −B and B = {0.10, 0.30, 0.60, 1.00}. All scenarios were repeated under the null- and alternative-hypotheses setting θ={0.00,0.05}. The BMRE estimator was evaluated using the following hyperparameters: $\mu_{\varphi }\;=\;\left\{0,0.05,0.10,\;0.15\right\},\;\mu_{\theta }\;=\;0$, with every element of $\mu_{\varphi }$ evaluated with five different variance hyperparameters: $\sigma_{\mu_{\varphi }}^{2}\;=\;\left\{10,\;10^{-2},\;10^{-2.4},10^{-2.7},10^{-3}\right\}$, and $\sigma_{\mu_{\theta }}^{2}\;=\;10$.

**Table 1:** Simulation scenarios of a multi-SNP Mendelian randomization study, with potential pleiotropic effects (i.e. violation of the exclusion restriction assumption)^a^

**Parameters**	**Scenario I**	**Scenario II**	**Scenario III**	**Scenario IV**	**Scenario V**
	(no pleiotropy)	(pleiotropy)	(InSIDE violated)	(partial pleiotropy)	(partial pleiotropy and InSIDE violated)
Number of subjects n	1000	1000	1000	1000	1000
Number of SNPs J	20	20	20	20	20
Proportion of pleiotropic SNPs q	1.0	1.0	1.0	**{0.1, 0.2, 0.3, 0.4}**	**0.4**
Minor allele frequency $p_{j}$	0.30	0.30	0.30	0.30	0.30
Effect of $G_{j}$ on $U_{i}$$\left(\omega_{1j}\right)$	0.00	0.00	Unif(L,U)	0.00	Unif(L,U)
Lower limit of $\omega_{1j}$L			L=0.00		L=0B={0.10,0.30,0.60,1.0}, and
Upper limit of $\omega_{1j}\;U$			U=0.50		L=−BB={0.10,0.30,0.60,1.0}
Effect of $G_{j}$ on $X_{i}$$\left(\alpha_{j}\right)$	Unif(0.5,4)	Unif(0.5,4)	Unif(0.5,4)	Unif(0.5,4)	Unif(0.5,4)
Effect of $G_{j}$ on $Y_{i}$$\left(\varphi_{j}\right)$	0.00	Unif(0,0.2)	Unif(0,0.2)	Unif(0,0.2)	Unif(0,0.2)
Effect of $U_{i}$ on $X_{i}$$\left(\omega_{2}\right)$	1	1	1	1	1
Effect of $U_{i}$ on $Y_{i}$$\left(\omega_{3}\right)$	1	1	1	1	1
Effect of $X_{i}$ on $Y_{i}$(θ)	{0.00, 0.05}	{0.00, 0.05}	{0.00, 0.05}	{0.00, 0.05}	{0.00, 0.05}

^a^Changes from the previous scenario (to the left) are presented in bold.

### Performance metrics

Performance was evaluated using the following metrics: bias defined as $\overset{¯}{\theta }-\theta$, with $\overset{¯}{\theta }$ equal to the mean of $\hat{\theta }$; the root mean square error $RMSE\;=\;\sqrt{bias^{2}+ESE^{2}},$ with ESE equal to the empirical standard error of $\hat{\theta }$: the proportion of rejected null-hypotheses (i.e. depending on whether θ equals 0 this is the type 1 error or power, using an alpha of 0.05).

All simulations were repeated 5000 times, with analyses performed using the statistical package R version 3.1.2 for Unix.^17^ The number of replications was chosen to ensure sufficient precision to detect small deviations from the nominal type 1 error rate of 0.05 (the 95% lower and upper bounds were 0.044 and 0.056). 

## Results

### Results of the simulation study

**Figure 1:**
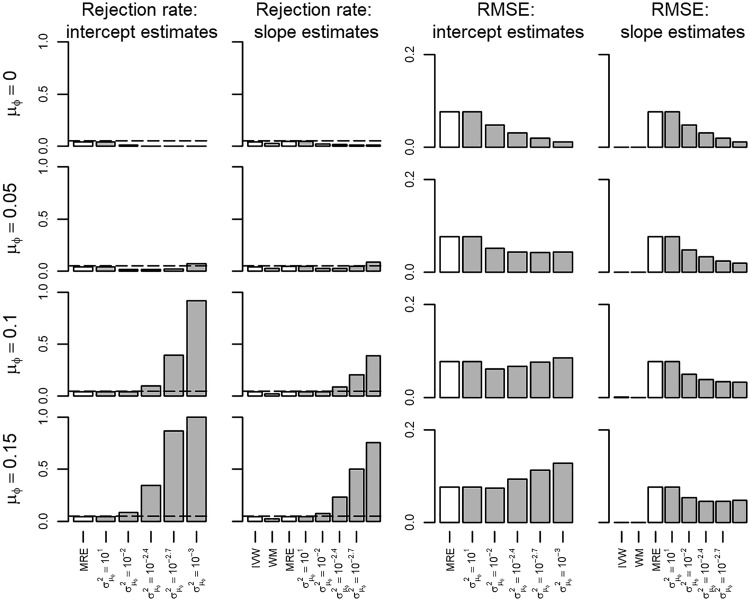
Rejection rate and root mean squared error of a Mendelian randomization study (scenario I) with the true slope of 0 and no unbalanced pleiotropy. IVW, inverse variance weighted; WM, weighted median; MRE, MR-Egger; $\mu_{\varphi }$ indicates the prior mean, and $\sigma_{\mu_{\varphi }}^{2}$ the prior variance of a Bayesian MRE. The underlying numerical values are presented in Appendix 3, available as Supplementary data at *IJE* online.

**Figure 2:**
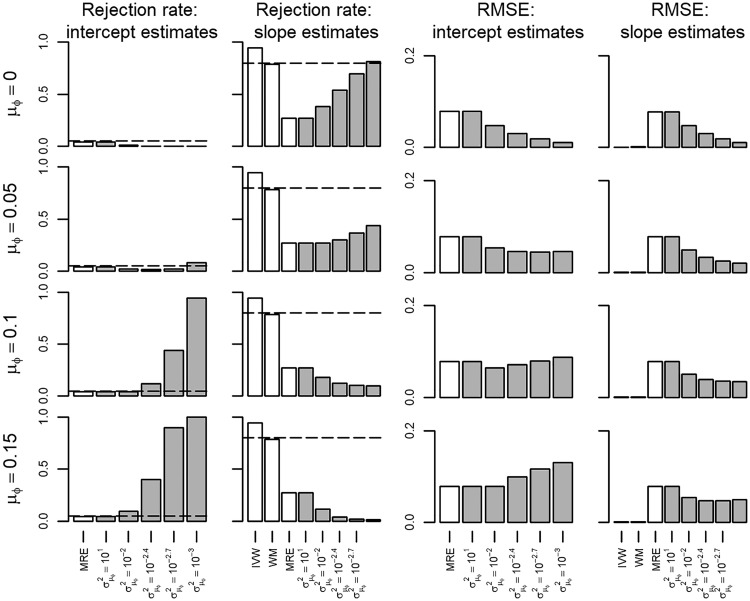
Rejection rate and root mean squared error of a Mendelian randomization study (scenario I) with the true slope of 0.05 and no unbalanced pleiotropy. IVW, inverse variance weighted; WM, weighted median; MRE, MR-Egger; $\mu_{\varphi }$ indicates the prior mean, and $\sigma_{\mu_{\varphi }}^{2}$ the prior variance of a Bayesian MRE. The underlying numerical values are presented in Appendix 3, available as Supplementary data at *IJE* online.

In scenario I all the MR assumptions held, hence all the IVW, WM and MRE estimators were unbiased (Appendix Figures 1–2, available as Supplementary data at *IJE* online). Bias of the BMRE estimates was minimal for the hyperparameters $\mu_{0}\;=\;\{0.00,\;0.05\}$, irrespective of the variance hyperparameter. Type 1 error rates of both the intercept and the slope estimates were generally below 0.05 using the same priors, and the RMSE markedly decreased with smaller values of $\sigma_{\mu_{\varphi }}^{2}$ (Figure 1). Repeating scenario 1 with the true slope set to 0.05, revealed that power of the BMRE estimator (relative to the MRE) was increased without increasing the intercept type 1 error rate above 0.05, unless $\mu_{\varphi }\geq 0.1$ and then only for small values of $\sigma_{\mu_{\varphi }}^{2}$ (Figure 2).

**Figure 3:**
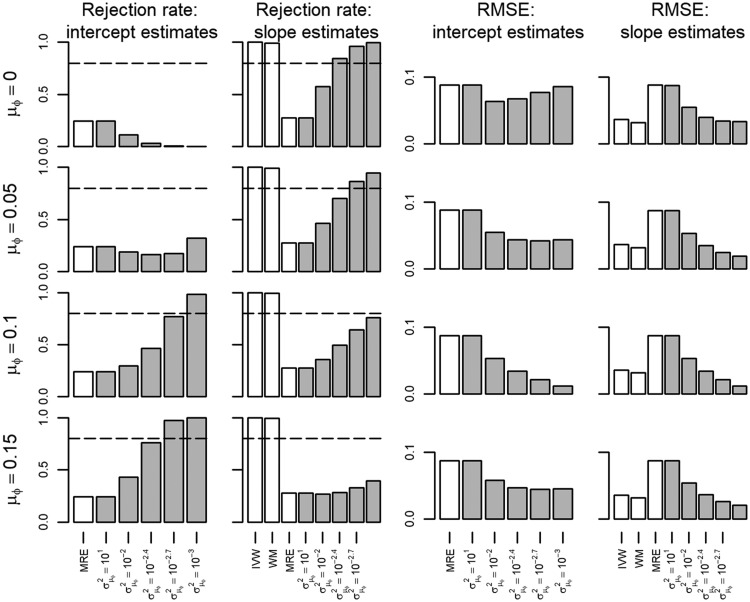
Rejection rate and root mean squared error of a Mendelian randomization study (scenario II) with the true slope of 0.05 and unbalanced pleiotropy. IVW, inverse variance weighted; WM, weighted median; MRE, MR-Egger; $\mu_{\varphi }$ indicates the prior mean, and $\sigma_{\mu_{\varphi }}^{2}$ the prior variance of a Bayesian MRE. The underlying numerical values are presented in Appendix 3, available as Supplementary data at *IJE* online.

Scenario II explored performance in the presence of pleiotropy which biased the IVW estimates, and (because 100% of the SNPs were pleiotropic) the WM. The MRE estimator remained unbiased (Appendix Figures 5–6, available as Supplementary data at *IJE* online), with the BMRE showing a similar pattern of bias as before, with bias depending on the size of $\sigma_{\mu_{\varphi }}^{2}$ when $\mu_{\varphi }\neq 0.10.\;$Intercept rejection rates (power) were increased when $\mu_{\varphi }\geq 0.10$ and $\sigma_{\mu_{\varphi }}^{2}\neq 10^{1}$; slope rejection rates (type 1 error) were close to nominal for all BMRE using $\sigma_{\mu_{\varphi }}^{2}\leq 10^{2.4}$ (Appendix Figure 7, available as Supplementary data at *IJE* online). In the same scenario (Appendix Figure 7) the type 1 error rates of the IVW estimator, and (to a lesser extent) the WM estimator, were inflated, at 0.73 and 0.44 respectively. Setting the phenotype effect to 0.05 (Figure 3) showed that power of the slope estimate was improved even when $\mu_{\varphi }$ was misspecified (i.e. not 0.10). Throughout the RMSE of the BMRE, estimators were equal to or lower than for the MRE estimator.

**Figure 4:**
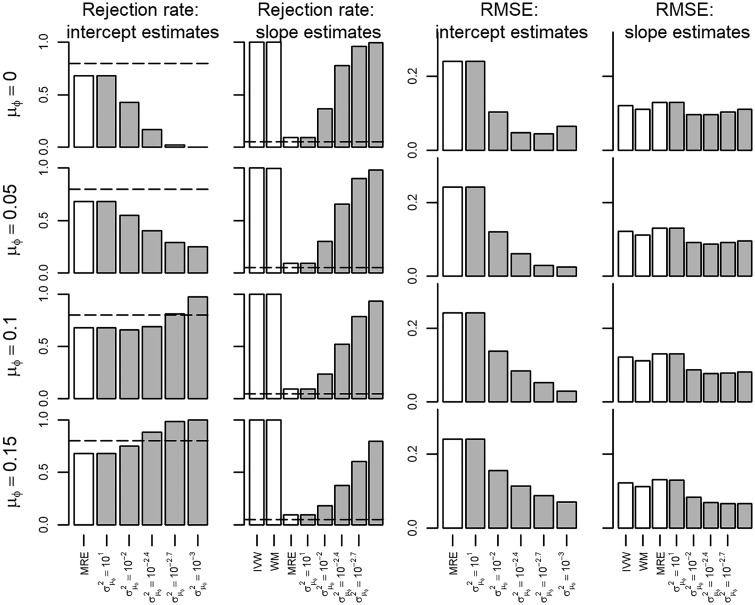
Rejection rate and root mean squared error of a Mendelian randomization study (scenario III) with the true slope of 0.05, and InSIDE assumption violated. IVW, inverse variance weighted; WM, weighted median; MRE, MR-Egger; $\mu_{\varphi }$ indicates the prior mean, and $\sigma_{\mu_{\varphi }}^{2}$ the prior variance of a Bayesian MRE. The underlying numerical values are presented in Appendix 3, available as Supplementary data at *IJE* online.

The InSIDE assumption was violated in scenario III which biased all estimators, with the more informative BMRE faring similarly to the IVW or WM estimators (Appendix Figures 9–10, available as Supplementary data at *IJE* online). Whereas the type 1 error rates of the IVW and WM estimators were close to 100%, the BMRE rejection rates depended on $\sigma_{\mu_{\varphi }}^{2}$ and often less than the IVW or WM methods (Figure 4). The MRE estimator had only slightly inflated type 1 error rates close to 0.05. The bias and inflated type 1 error rate of the BMRE persisted even when the intercept prior mean was correctly specified at 0.10 (Figure 4). In these settings, the BMRE estimator was generally more powerful than the MRE approach, which is of limited value given the observed bias and inflated type 1 error rates (Appendix Figure 12, available as Supplementary data at *IJE* online).

**Figure 5:**
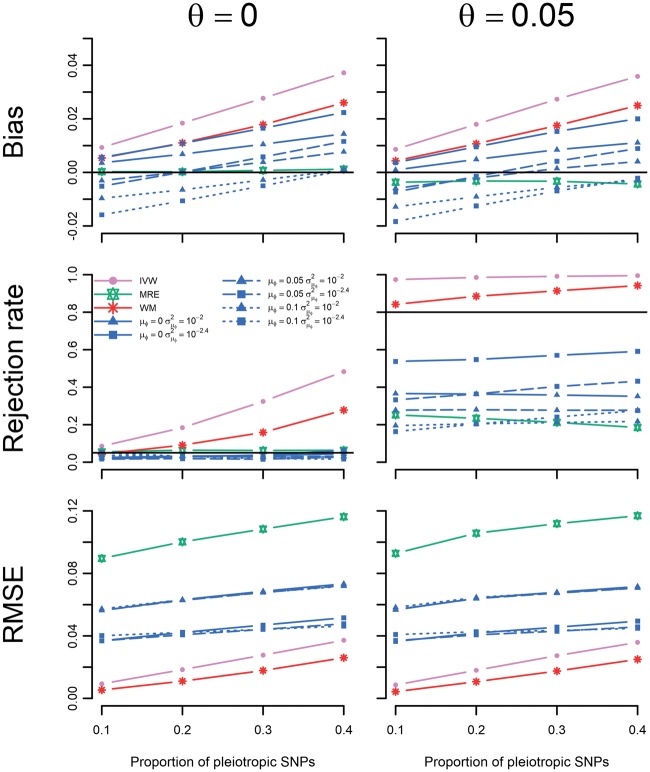
Simulation results of scenario IV: the causal effect estimated in Mendelian randomization study with different proportions of pleiotropic SNPs. IVW, inverse variance weighted; WM, weighted median; MRE, MR-Egger; $\mu_{\varphi }$ indicates the prior mean, and $\sigma_{\mu_{\varphi }}^{2}$ the prior variance of a Bayesian MRE. The underlying numerical values are presented in Appendix 3, available as Supplementary data at *IJE* online.

**Figure 6:**
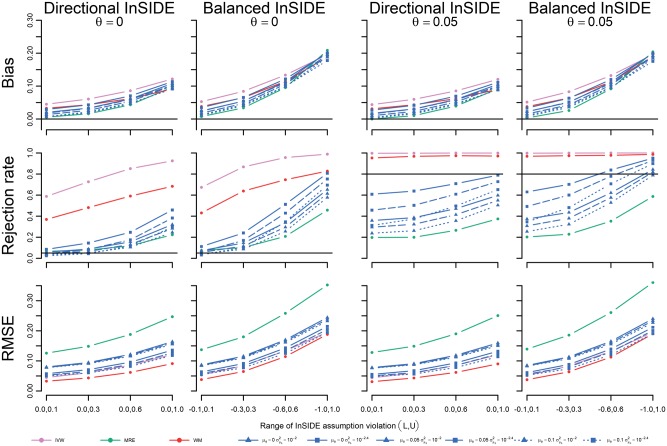
Simulation results of scenario V: the causal effect estimated in a Mendelian randomization study with 40% pleiotropic SNPs and different degrees of InSIDE assumption violation; left panel: no causal effect; right panel: causal effect of 0.05. IVW, inverse variance weighted; WM, weighted median; MRE, MR-Egger; $\mu_{\varphi }$ indicates the prior mean, and $\sigma_{\mu_{\varphi }}^{2}$ the prior variance of a Bayesian MRE. The underlying numerical values are presented in Appendix 3, available as Supplementary data at *IJE* online.

The performance of these estimators was further explored in scenario IV by varying the proportion of pleiotropic SNPs. The BMRE results focused on the previously optimally performing combinations of hyperparameters: $\mu_{\varphi }=\left\{0,0.05,0.10\right\},\;\sigma_{\mu_{\varphi }}^{2}=\left\{\;10^{-2},\;10^{-2.4}\right\}$. Note that in this and the next scenario, the average pleiotropy depends on the proportion of pleiotropic SNPs, which ranged between 0.025 (for 10% invalid SNPs) and 0.100 (for 40% invalid SNPs), resulting in differing levels of BMRE misspecification. Figure 5 shows the MRE to be the only unbiased estimator in this scenario. Type 1 error rates were inflated for the IVW and WM methods, with power of the BMRE approach typically surpassing that of the MRE estimator. Next in scenario V, we explored the impact of different degrees of InSIDE assumption violation, revealing a similar amount of bias for all estimators (Figure 6). Type 1 error rates and power were general highest for the IVW, (closely) followed by WM, the BMRE and MRE methods. As before, the MRE had the largest RMSE throughout, with smaller values for the BMRE, IVW and the WM estimators.

### Prior elucidation using empirical data

To illustrate the proposed BMRE method and provide an example of how to elucidate a sensible prior distribution, we consider the study by White *et al.*^7^ This study explored the relation between urate and CHD using 31 SNPs collected from 166 486 individuals, 9784 of whom had CHD. White and colleagues used both the IVW and the MRE methods, which showed conflicting results: odds ratio (OR) 1.18 (95% confidence interval (CI) 1.03; 1.34) for the IVW estimate compared with OR 1.05 (95% CI 0.87; 1.27) for the MRE estimate; both re-calculated here using a pleiotropy robust multiplicative random effects model. Aside from the difference in point estimate, the MRE estimate is considerably more variable (standard error (se) of 0.096, compared with an IVW se of 0.066), resulting in wide confidence interval bounds. Interestingly the MRE pleiotropy (intercept) OR estimate of 1.008 (95% CI 0.998; 1.018) is precise, seemingly indicating that amount of directional pleiotropy is minimal, thus questioning the necessity of a MRE pleiotropy correction. In the following, we will explore the utility of the BMRE to increase precision of the slope estimate and further explore the necessity of the pleiotropy correction.

White and colleagues not only collected data on CHD and urate, but also on many potential pleiotropic pathways (Appendix Figure 13, available as Supplementary data at *IJE* online) allowing a thorough exploration of the magnitude and direction of observed pleiotropy. We note that four SNPs (rs1260326, rs3741414, rs1178977, rs653178) show clear pleiotropic signals (based on a genome-wide significant *P*-value). Given the number of candidate SNPs, it would be sensible to exclude these SNPs; however, to illustrate the utility of the BMRE we will include these SNPs. Inclusion of pleiotropic SNPs may also occur in practice, for example, when the number of candidate SNPs is modest. Additionally, there is no a priori reason to assume pleiotropy is limited to genome-wide significant signals, hence exclusion of these four SNPs will not necessarily remove all (or even most) of the pleiotropy.

To elucidate and model the likely (known and unknown) pleiotropic effects, we plot the SNP associations with the different phenotypes (Appendix Figure 13), which shows a symmetrical (balanced) pattern centred on a null effect, with most of the estimates between ± 0.05. Although reassuring, this does not preclude the possibility of unobserved pleiotropy via different pathways. Based on the observed pleiotropy effects (Appendix Figure 13), we set the mean prior hyperparameter to $\mu_{\varphi }\;=\;0.00$ and considered the following prior variance hyperparameters: $\sigma_{\mu_{\varphi }}^{2}\;=\;\left\{10^{-6},\;10^{-5.8},\;10^{-5.6},\;10^{-5.4},\;10^{-5.2}\right\}$. These values of the prior variance parameters were chosen to initially approximate the IVW estimator, incrementally including more uncertainty and thereby allowing for additional pleiotropy. Second, in an alternative approach we use the empirical data to also elucidate the prior variance hyperparameter by selecting a prior variance $\sigma_{\mu_{\varphi }}^{2}\;=6.508\times 10^{-4}∼10^{-3.2}$, putting approximately 95% of the prior distribution ± 0.05 (the range containing most of the observed pleiotropy signals).

Results of the first approach are shown in Table 2, with the BMRE method showing larger slope estimates (OR ranges from 1.17 to 1.13), than the attenuated MRE OR estimate of 1.05 and the WM 1.12 (95% CI 0.99; 1.27). The BMRE credible intervals included the neutral value of 1 at a prior variance of $10^{-5.6}$; under this prior the intercept odds has 95% probability of lying in (0.997,1.003) suggesting that the balanced pleiotropy assumption has a relevant impact on our IV estimates. Similarly when using the empirically elucidated variance hyperparameter of $6.508\times 10^{-4},$ the BMRE slope estimate becomes OR 1.05 (95% CI 0.87; 1.27) which is identical (to 2 dp) to the MRE estimate. Using the BMRE method we can thus confidently say that despite the empirical data showing balanced pleiotropy, and the tight confidence interval around the MRE intercept estimate, there is relevant directional pleiotropy and the pleiotropy-corrected estimates should be preferred over the IVW estimate.

**Table 2:** Results of a Mendelian randomization study on the effect of plasma urate on CHD with different IV estimators

	**Estimates**	
	Intercept	Slope
	OR (95% CI)	OR (95% CI)
IVW		
		1.18 (1.03; 1.34)
MRE		
	1.008 (0.998; 1.018)	1.05 (0.87; 1.27)
BMRE		
$\sigma_{\varphi }^{2}\;=\;10^{-6}$	1.001 (0.998; 1.003)	1.17 (1.02; 1.34)
$\sigma_{\varphi }^{2}\;=\;10^{-5.8}$	1.001 (0.998; 1.004)	1.16 (1.01; 1.34)
$\sigma_{\varphi }^{2}\;=\;10^{-5.6}$	1.001 (0.997; 1.006)	1.15 (1.00; 1.33)
$\sigma_{\varphi }^{2}\;=\;10^{-5.4\;}$	1.002 (0.997; 1.007)	1.14 (0.99; 1.33)
$\sigma_{\varphi }^{2}\;=\;10^{-5.2\;}$	1.003 (0.997; 1.009)	1.13 (0.97; 1.32)
WM		1.12 (0.99; 1.27)

Results presented as odds ratio per 1 SD increase in urate with 95% confidence (or credibility) interval (CI) in brackets. The intercept measures the amount of pleiotropy, the slope estimates the effect of plasma urate on CHD. IVW, inverse variance weighted method; MRE, MR-Egger method; BMRE, Bayesian MR-egger method; WM, weighted median method. $\mu_{\varphi }\;=\;0$, the slope mean and variance priors were 0 and 10 throughout, respectively.

## Discussion

In this paper, we introduce a novel Bayesian implementation of the MR-Egger (BMRE) method for instrumental variable analyses, robust to violation of the exclusion restriction assumption due to pleiotropy. We show that under the InSIDE assumption, the BMRE estimator with weakly informative priors on the intercept increases power to detect a causal effect, while retaining acceptable type 1 error rates. Additionally, the root mean square error of the BMRE estimator was lower than that of the traditional MRE method and, in the presence of pleiotropy, lower than the IVW estimator. Using the empirical example of urate and CHD, we present an approach to evaluate and elucidate sensible prior parameters for the presence of pleiotropy.

When the InSIDE assumption was violated, all estimators were biased and showed inappropriately high rejection rates. In this case, adding prior belief increased bias and rejection rates of the BMRE towards those of the IVW estimator. Comparing the BMRE with the WM method showed that (depending on the prior) the BMRE approach had lower type 1 error rates and was more robust to different degrees of InSIDE assumption violation. Furthermore, if 100% of the SNPs were pleiotropic, the BMRE approach generally was less biased, with type 1 error rates closer to nominal than the WM estimator. In the presence of InSIDE assumption violation, the MRE estimator performed better than the BMRE method. The InSIDE assumption may be violated in empirical data, for example when the pleiotropy effects of different variants affect the outcome via the same set of confounders. Pickrell *et al.*, however, present evidence that pleiotropic SNPs often work via independent pathways, suggesting the InSIDE assumption may hold more generally.^18^

The analyses presented here are naturally limited and the following deserves consideration. First, we chose to implement the BMRE using conjugate priors because these have closed form solutions which increase ease of use and provide exact solutions. In most empirical settings, conjugate priors seem sufficient and are a natural way to encode prior knowledge. Furthermore, the normal distribution is not sharply peaked at its mean value, allowing a reasonable range of values to be given high prior probability, while still discounting unreasonably large values. Second, whereas the IVW method is susceptible to directional pleiotropy, this estimator generally has more precision and power and is more robust to uncertainty in the SNP-exposure association.^19^ As such, the IVW method should, in our opinion, remain the starting point of any MR analysis, with other approaches including the WM, MRE and BMRE used as informative sensitivity analyses. Third, the BMRE methods were evaluated on frequentist concepts of power and type 1 error. Given that MR analyses are often used to test whether a biomarker has a causal effect on disease, we feel these metrics are relevant. Fourth, whereas the weakly informative hyperparameter of $\mu_{\varphi }=\{0.00,0.05\}$ and $\sigma_{\mu_{\varphi }}^{2}\leq 10^{-2.4}$ had the desired property of increasing power while maintaining type 1 error rates close to nominal, this is specific to the scenarios considered. Indeed, as we show in our empirical example, these prior hyperparameters should be tailored to the data under consideration. We encourage empirical researchers to use our example as a blueprint to explore observed pleiotropy and to tailor the hyperparameters. In practice, analyses should be repeated under a range of variance hyperparameters, to gain a sense of how precise the prior beliefs must be to maintain significant evidence of causality. Additionally, and similar to designing a Bayesian randomized controlled trial, one may wish to repeat the simulations using scenarios based on the available empirical data and explore performance (see Appendix 2 for the simulation code which took 42 s to run 500 replications of scenario II).

The BMRE method can be used to explore the importance of the balanced pleiotropy assumption of the IVW estimator, and may be particularly useful for reconciling conflicting results from IVW and MRE methods, as we have shown in our example of urate and CHD. Applied researchers may wish to look to a recent framework^14^ reviewing the underlying assumptions of the IVW and MRE methods, as well as describing a number of goodness-of-fit statistics and sensitivity analyses. By using a conjugate Bayesian prior, the same framework can readily be applied to the BMRE method presented here. Similarly, the SIMEX^19^ adjustment for uncertainty in the SNP-exposure association can be readily applied to our BMRE method as well.

In addition to MRE and WM methods, several other approaches to deal with pleiotropy have recently been proposed, each with its own assumptions, including a weighted mode estimator^20^ and a Bayesian model averaging^21^ approach conceptually similar to ours. Furthermore, detection and removal of SNPs yielding outlier IV estimates is an important step that can be combined with the pleiotropy robust estimators.^14^ A full comparison of methods under realistic settings is beyond the scope of this paper, but a sensible strategy in general is to perform a series of complementary sensitivity analyses in addition to the standard IVW analysis. In this regard, our BMRE method can increase the precision of the MRE estimator and provide insight into discrepancies between IVW and MRE analyses. Further, our BMRE method may be especially useful when candidate instruments show likely pleiotropic effects, but there are too few strong instruments to exclude these pleiotropic SNPs.

In conclusion, we introduce a Bayesian version of the MR-Egger method, which, by placing weakly informative priors on the intercept term increases power over MR-Egger while retaining acceptable type 1 error rates. Violations of the InSIDE assumption increase bias and type 1 error rates beyond those of the MR-Egger method. We suggest that Bayesian MR-Egger is a useful sensitivity analysis that can strengthen the evidence for causal effects in MR studies, particularly in the presence of observed pleiotropy. 

## Funding

A.F.S. is funded by UCLH NIHR Biomedical Research Centre and is a UCL Springboard Population Health Sciences Fellow. F.D. is funded by the MRC (K006215).

## Supplementary Material

Supplementary Data
