# Supplementary material for: Mendelian randomization with Egger pleiotropy correction and weakly informative Bayesian priors
Source: Int J Epidemiol. 2017 Dec 15;47(4):1217–28. doi: 10.1093/ije/dyx254 (PMC6124638; doi:10.1093/ije/dyx254)
Supplement: Supplementary Data [file dyx254_supp.zip › dyx254-suppl_data/ije-2017-02-0146-File009.pdf]

Mendelian randomization with Egger pleiotropy correction and weakly informative Bayesian priors:

Appendix figures

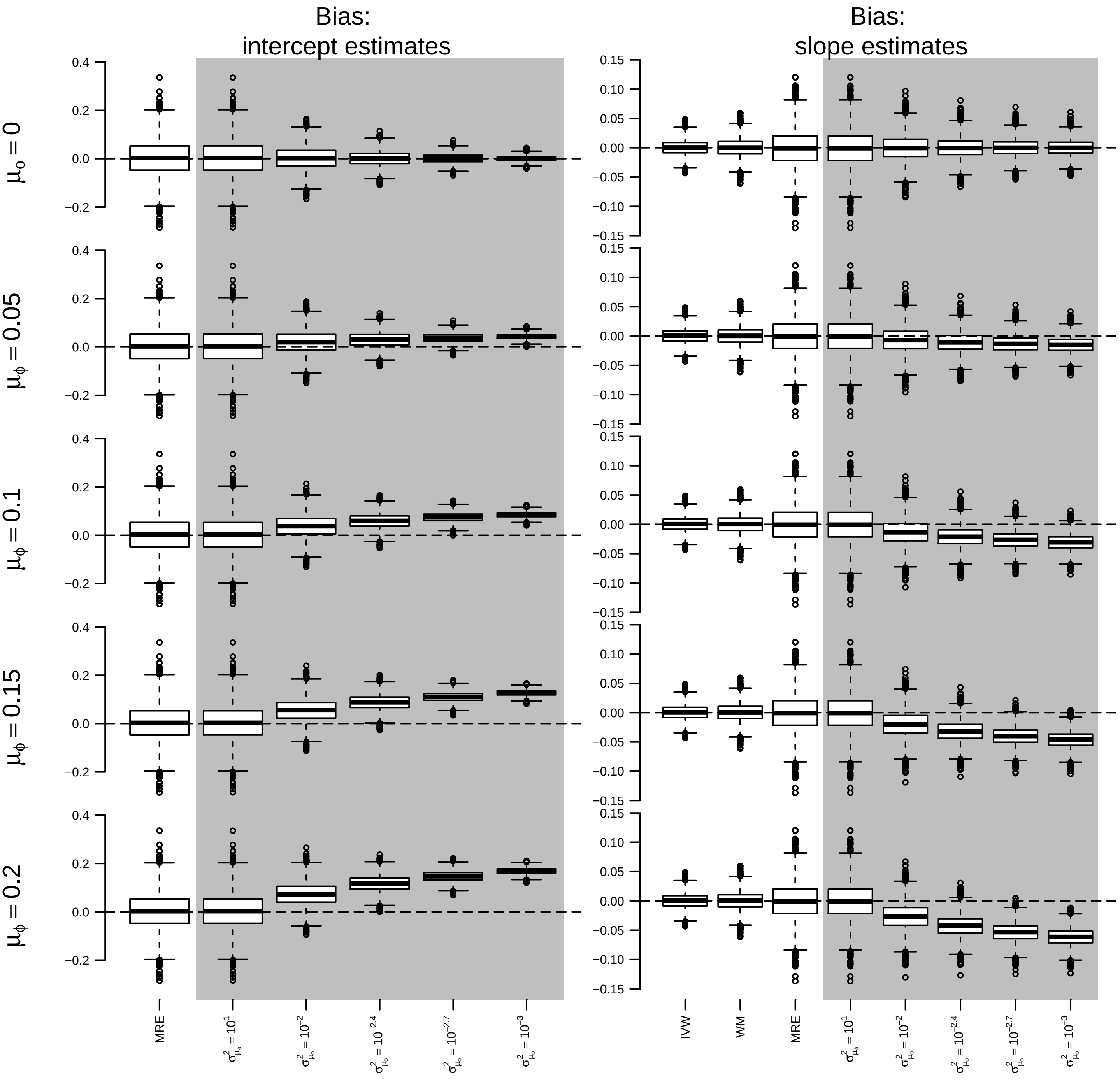

Figure 1: Bias of a Mendelian randomization study using instrumental variable analyses with and without weakly informative Bayesian priors on the intercept (pleiotropy) term. Scenario I without pleiotropy and no effect of the phenotype on the outcome; $\phi_j = 0.00$ and $\theta = 0.00$, with J 20 independent SNPs, sampled from $n = 1,000$ subjects, and 5,000 replications. IVW = inverse variance weighted instrumental variable analysis without Egger correction; WM = Weighted Median estimator; MRE = Mendelian Randomization study using an instrumental variable analysis with Egger correction; μ_ϕ and $\sigma^2_{\mu_\phi}$ are the prior hyperparameters for the mean and variance of the Bayesian MRE.

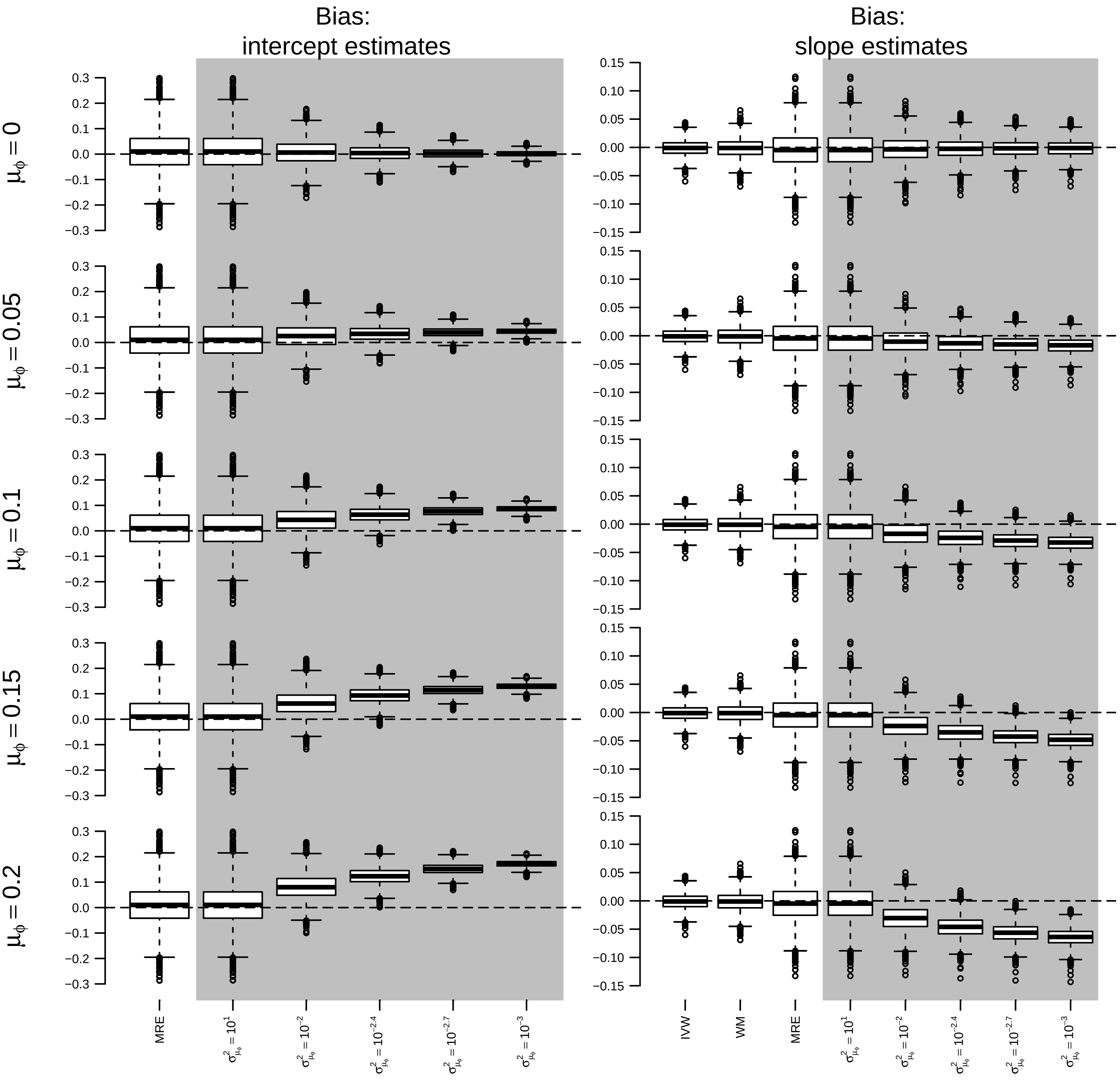

Figure 2: Bias of a Mendelian randomization study using instrumental variable analyses with and without weakly informative Bayesian priors on the intercept (pleiotropy) term. Scenario I without pleiotropy and a causal effect of the phenotype on the outcome; $\phi_j = 0.00$ and $\theta = 0.05$, with J 20 independent SNPs, sampled from $n = 1,000$ subjects, and 5,000 replications. IVW = inverse variance weighted instrumental variable analysis without Egger correction; WM = Weighted Median estimator; MRE = Mendelian Randomization study using an instrumental variable analysis with Egger correction; μ_ϕ and $\sigma^2_{\mu_\phi}$ are the prior hyperparameters for the mean and variance of the Bayesian MRE.

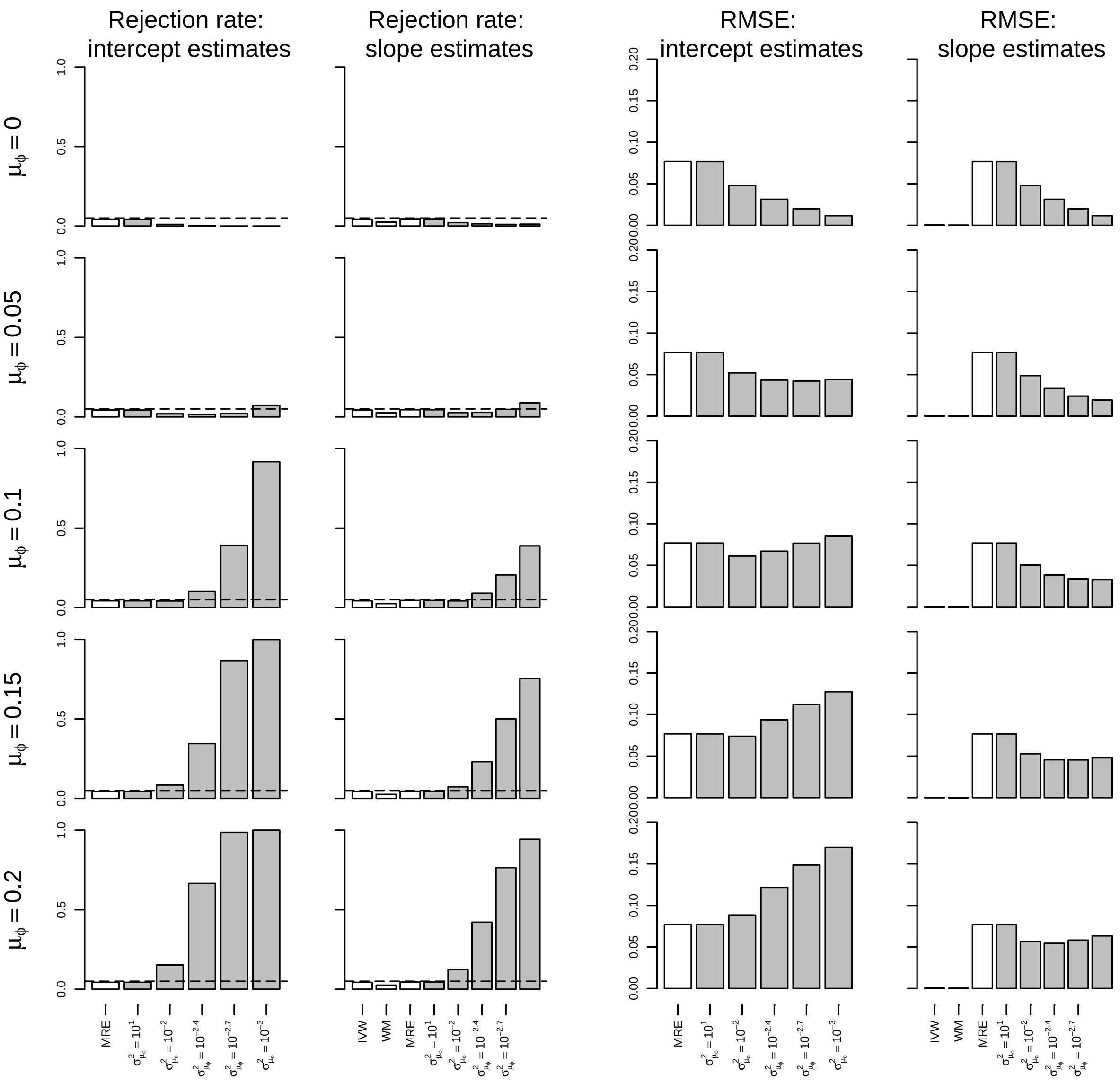

Figure 3: Rejection rate and root mean square error (RMSE) of a Mendelian randomization study using instrumental variable analyses with and without weakly informative Bayesian priors on the intercept (pleiotropy) term. Scenario I without pleiotropy and no effect of the phenotype on the outcome; $\phi_j = 0.00$ and $\theta = 0.00$, with J 20 independent SNPs, sampled from $n = 1,000$ subjects, and 5,000 replications. IVW = inverse variance weighted instrumental variable analysis without Egger correction; WM = Weighted Median estimator; MRE = Mendelian Randomization study using an instrumental variable analysis with Egger correction; μ_ϕ and $\sigma^2_{\mu_\phi}$ are the prior hyperparameters for the mean and variance of the Bayesian MRE.

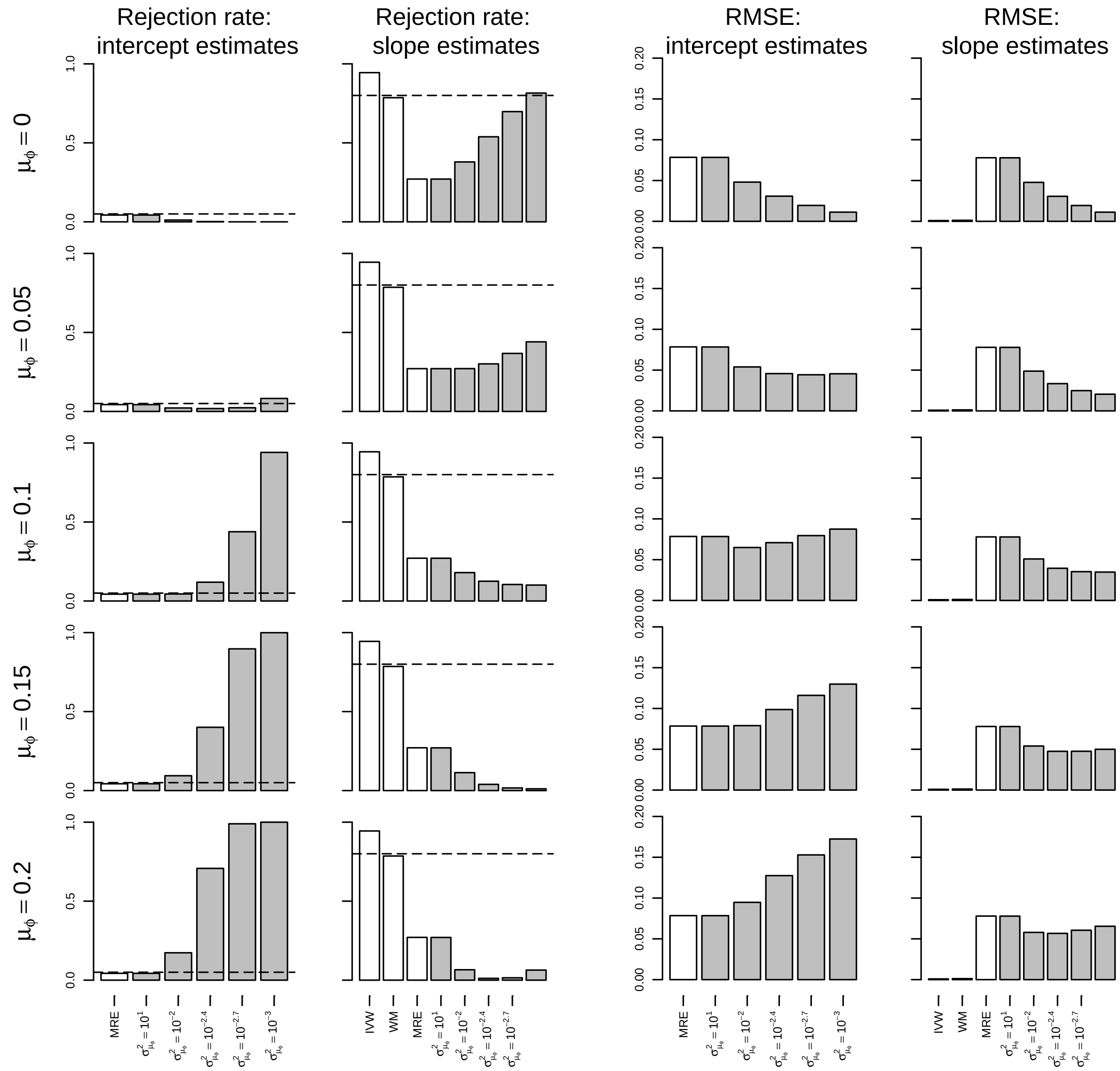

Figure 4: Rejection rate and root mean square error (RMSE) of a Mendelian randomization study using instrumental variable analyses with and without weakly informative Bayesian priors on the intercept (pleiotropy) term. Scenario I without pleiotropy and a causal effect of the phenotype on the outcome; $\phi_j = 0.00$ and $\theta = 0.05$, with J 20 independent SNPs, sampled from $n = 1,000$ subjects, and 5,000 replications. IVW = inverse variance weighted instrumental variable analysis without Egger correction; WM = Weighted Median estimator; MRE = Mendelian Randomization study using an instrumental variable analysis with Egger correction; μ_ϕ and $\sigma^2_{\mu_\phi}$ are the prior hyperparameters for the mean and variance of the Bayesian MRE.

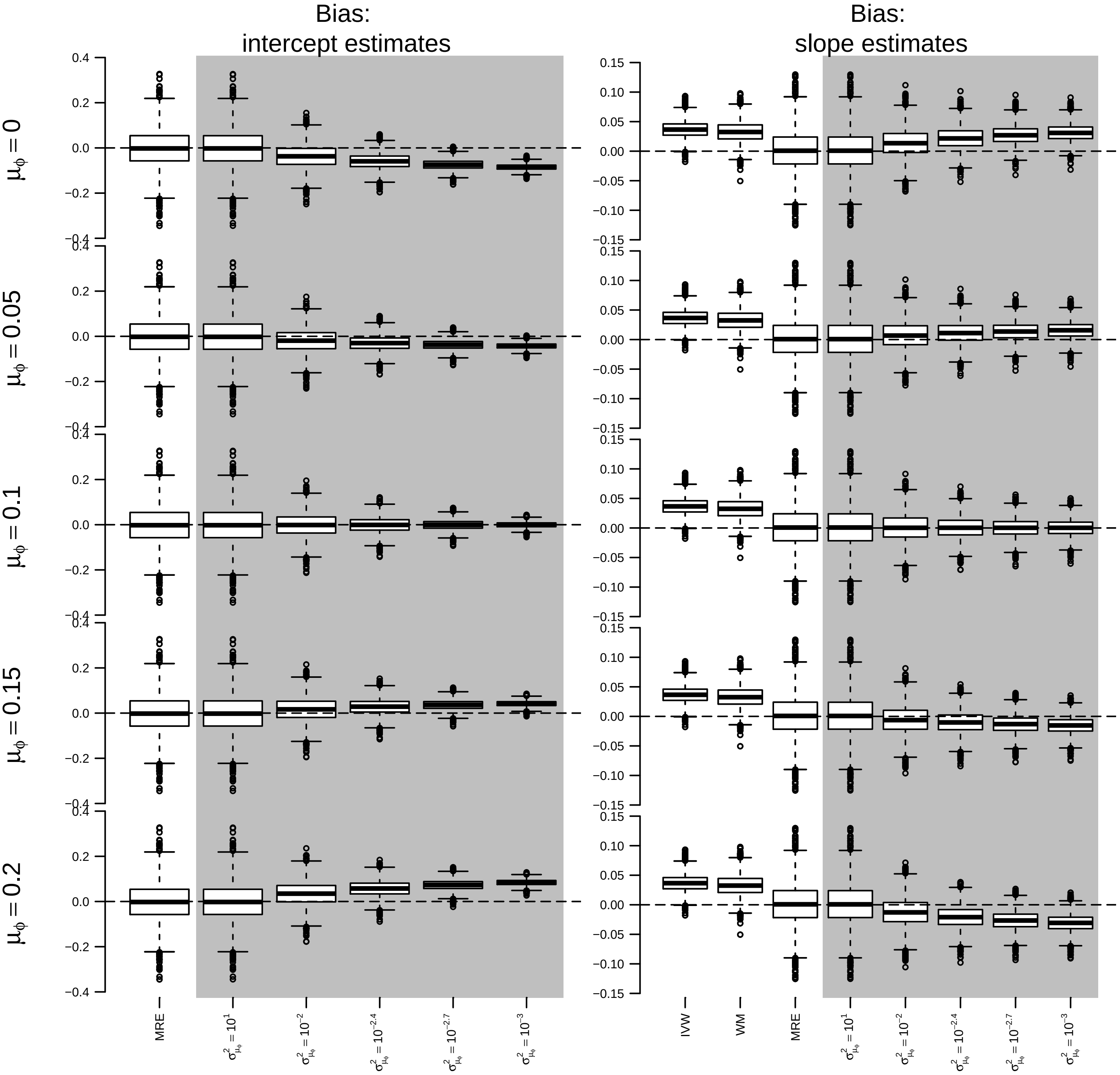

Figure 5: Bias of a Mendelian randomization study using instrumental variable analyses with and without weakly informative Bayesian priors on the intercept (pleiotropy) term. Scenario II with pleiotropy and no effect of the phenotype on the outcome; $\phi_j = 0.10$ and $\theta = 0.00$, with J 20 independent SNPs, sampled from $n = 1,000$ subjects, and 5,000 replications. IVW = inverse variance weighted instrumental variable analysis without Egger correction; WM = Weighted Median estimator; MRE = Mendelian Randomization study using an instrumental variable analysis with Egger correction; μ_ϕ and $\sigma^2_{\mu_\phi}$ are the prior hyperparameters for the mean and variance of the Bayesian MRE.

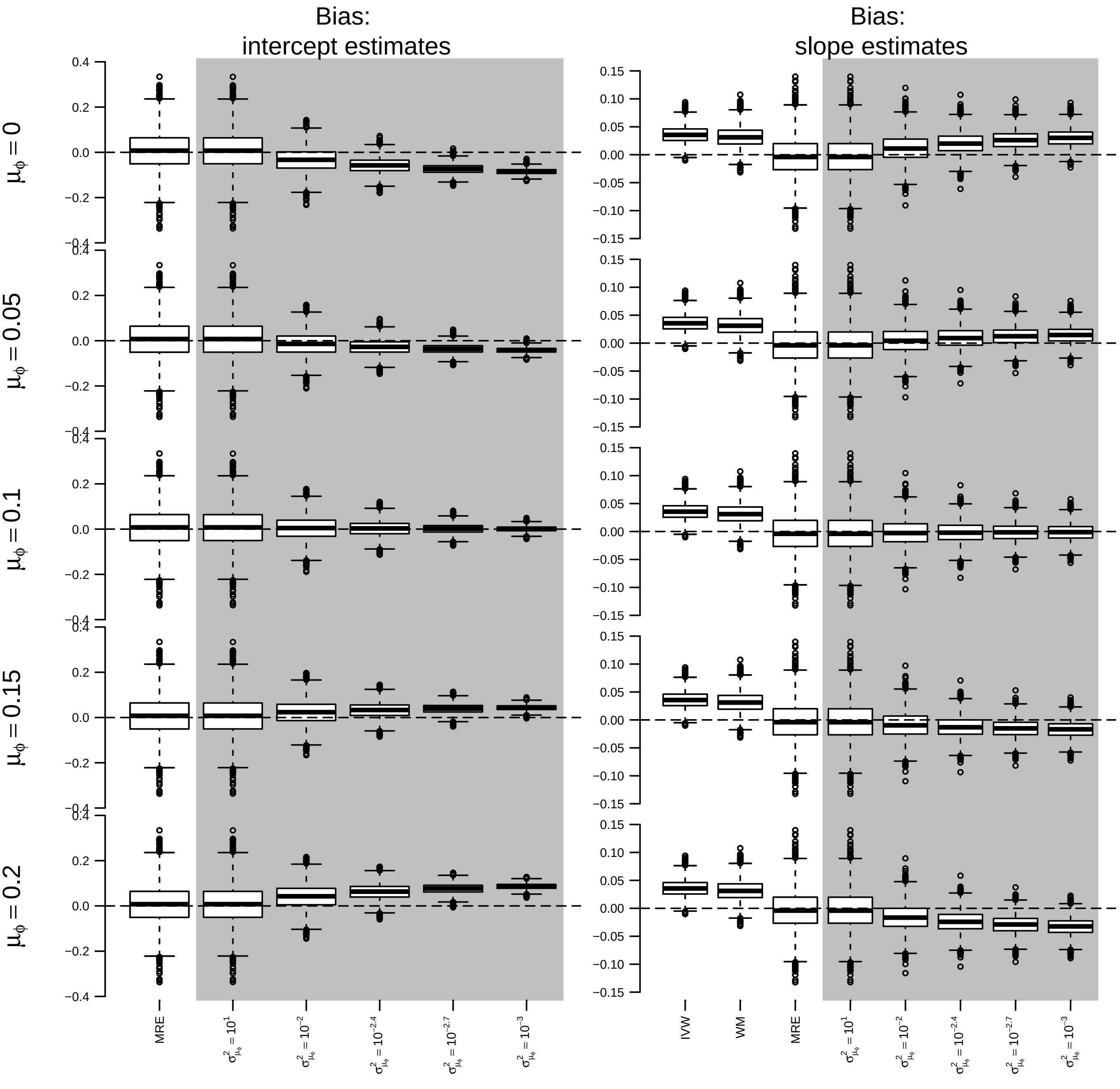

Figure 6: Bias of a Mendelian randomization study using instrumental variable analyses with and without weakly informative Bayesian priors on the intercept (pleiotropy) term. Scenario II with pleiotropy and a causal effect of the phenotype on the outcome; $\phi_j = 0.10$ and $\theta = 0.05$, with J 20 independent SNPs, sampled from $n = 1,000$ subjects, and 5,000 replications. IVW = inverse variance weighted instrumental variable analysis without Egger correction; WM = Weighted Median estimator; MRE = Mendelian Randomization study using an instrumental variable analysis with Egger correction; μ_ϕ and $\sigma^2_{\mu_\phi}$ are the prior hyperparameters for the mean and variance of the Bayesian MRE.

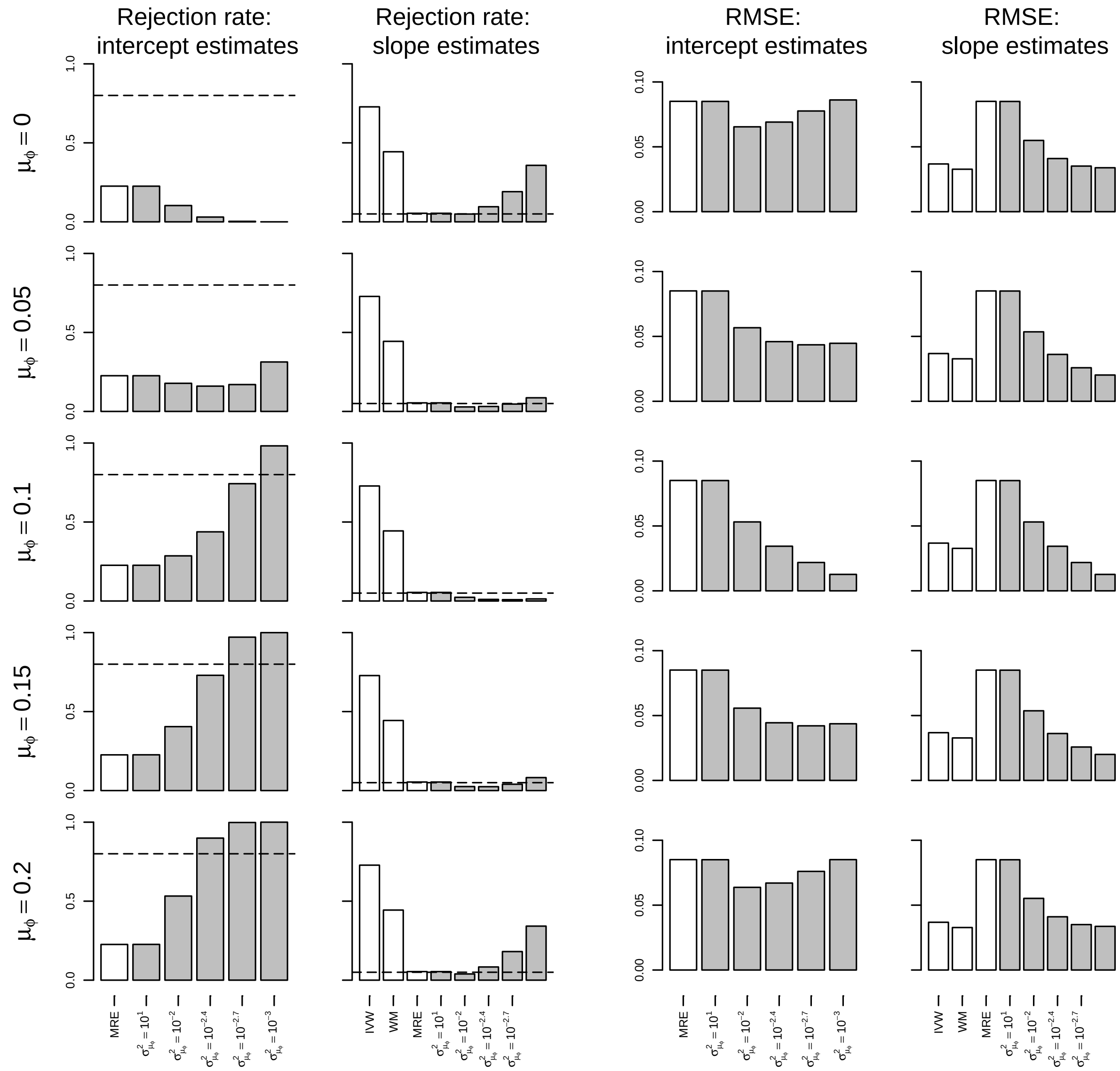

Figure 7: Rejection rate and root mean square error (RMSE) of a Mendelian randomization study using instrumental variable analyses with and without weakly informative Bayesian priors on the intercept (pleiotropy) term. Scenario II with pleiotropy and no effect of the phenotype on the outcome; $\phi_j = 0.10$ and $\theta = 0.00$, with J 20 independent SNPs, sampled from $n = 1,000$ subjects, and 5,000 replications. IVW = inverse variance weighted instrumental variable analysis without Egger correction; WM = Weighted Median estimator; MRE = Mendelian Randomization study using an instrumental variable analysis with Egger correction; μ_ϕ and $\sigma^2_{\mu_\phi}$ are the prior hyperparameters for the mean and variance of the Bayesian MRE.

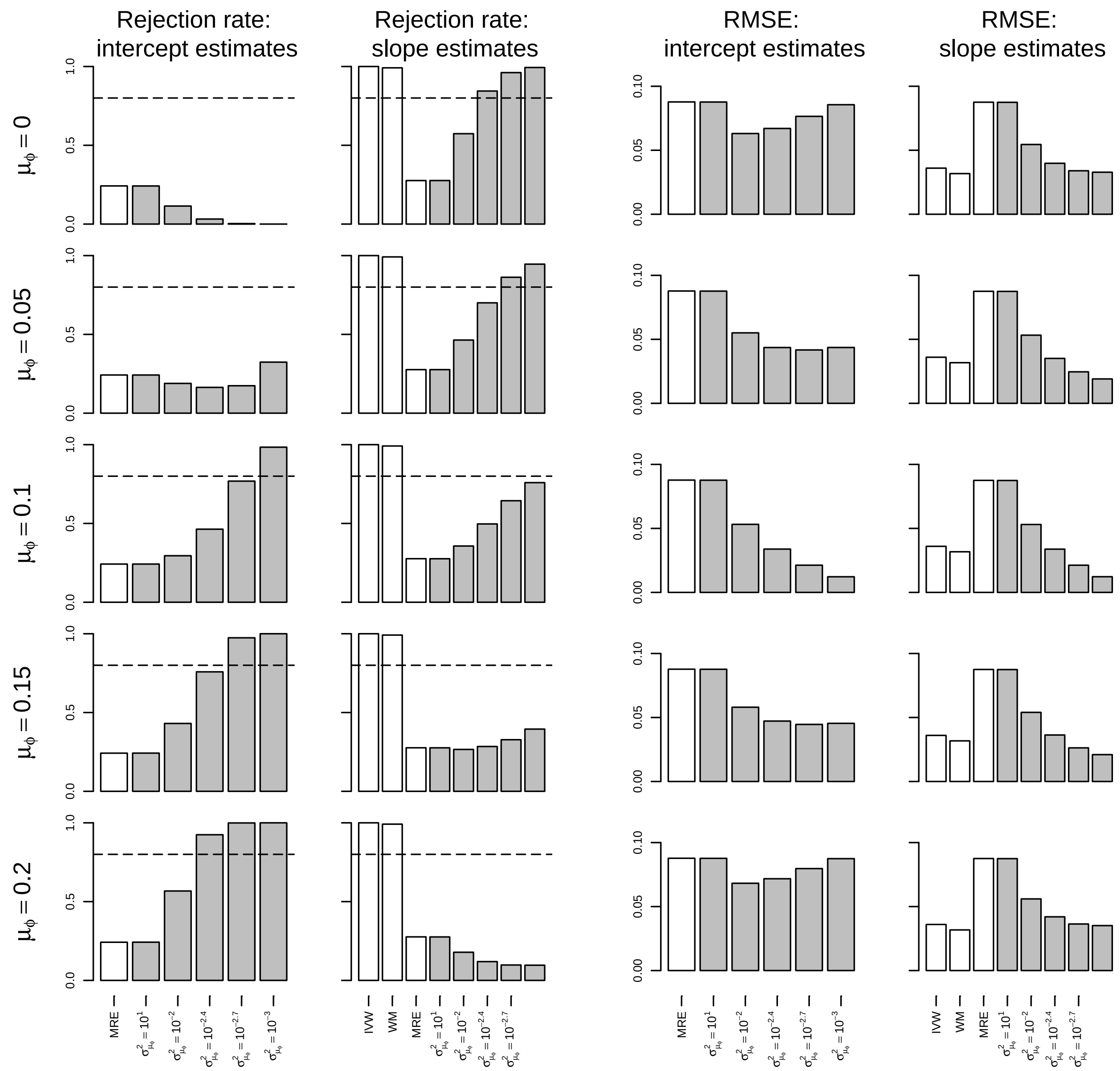

Figure 8: Rejection rate and root mean square error (RMSE) of a Mendelian randomization study using instrumental variable analyses with and without weakly informative Bayesian priors on the intercept (pleiotropy) term. Scenario II with pleiotropy and a causal effect of the phenotype on the outcome; $\phi_j = 0.10$ and $\theta = 0.05$, with J 20 independent SNPs, sampled from $n = 1,000$ subjects, and 5,000 replications. IVW = inverse variance weighted instrumental variable analysis without Egger correction; WM = Weighted Median estimator; MRE = Mendelian Randomization study using an instrumental variable analysis with Egger correction; μ_ϕ and $\sigma^2_{\mu_\phi}$ are the prior hyperparameters for the mean and variance of the Bayesian MRE.

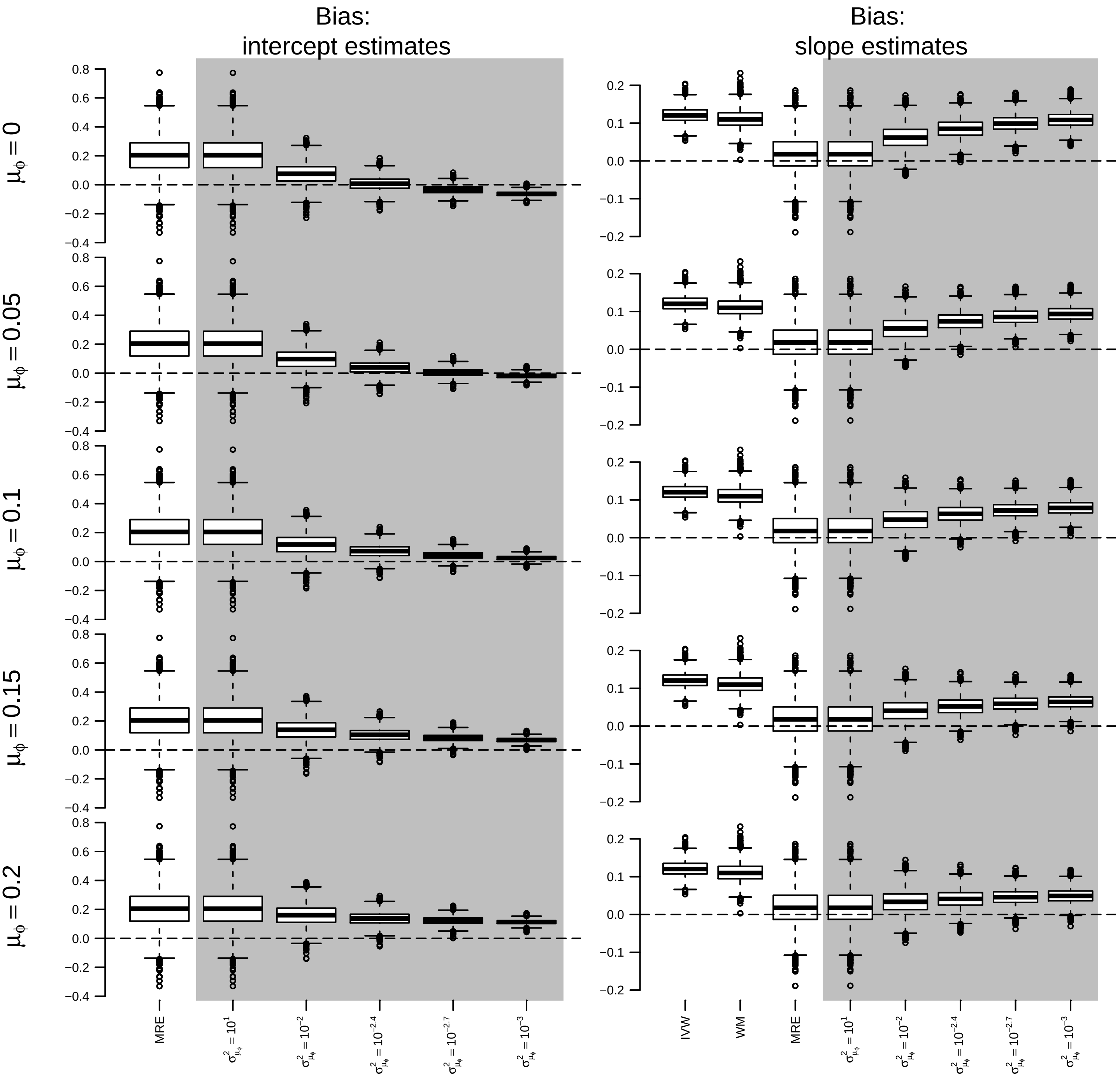

Figure 9: Bias of a Mendelian randomization study using instrumental variable analyses with and without weakly informative Bayesian priors on the intercept (pleiotropy) term. Scenario III with pleiotropy, the InSIDE assumption violated and no effect of the phenotype on the outcome; $\phi_j = 0.10$ and $\theta = 0.00$, with J 20 independent SNPs, sampled from $n = 1,000$ subjects, and 5,000 replications. IVW = inverse variance weighted instrumental variable analysis without Egger correction; WM = Weighted Median estimator; MRE = Mendelian Randomization study using an instrumental variable analysis with Egger correction; μ_ϕ and $\sigma^2_{\mu_\phi}$ are the prior hyperparameters for the mean and variance of the Bayesian MRE.

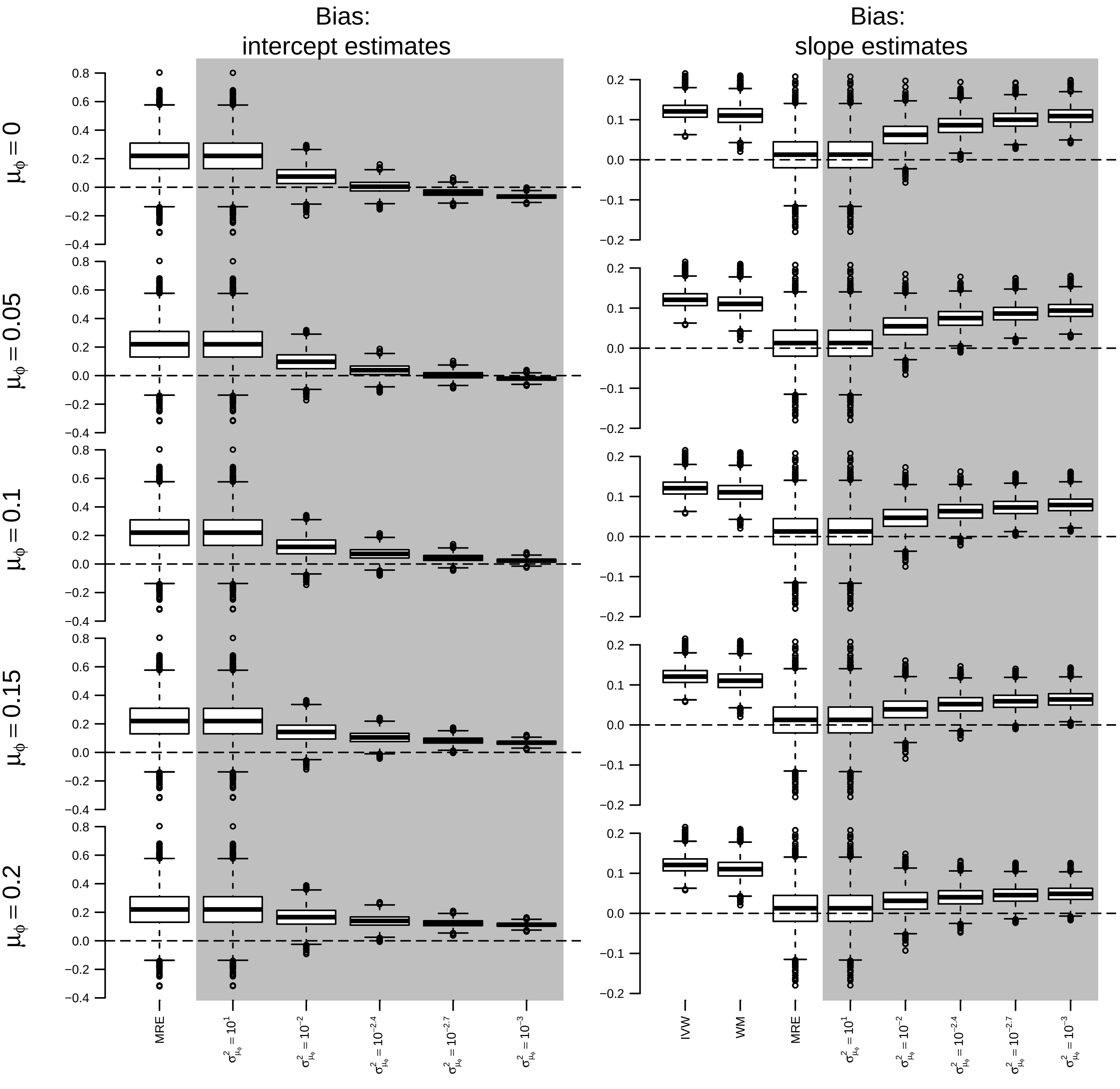

Figure 10: Bias of a Mendelian randomization study using instrumental variable analyses with and without weakly informative Bayesian priors on the intercept (pleiotropy) term. Scenario III with pleiotropy, the InSIDE assumption violated and a causal effect of the phenotype on the outcome; $\phi_j = 0.10$ and $\theta = 0.05$, with J 20 independent SNPs, sampled from $n = 1,000$ subjects, and 5,000 replications. IVW = inverse variance weighted instrumental variable analysis without Egger correction; WM = Weighted Median estimator; MRE = Mendelian Randomization study using an instrumental variable analysis with Egger correction; μ_ϕ and $\sigma^2_{\mu_\phi}$ are the prior hyperparameters for the mean and variance of the Bayesian MRE.

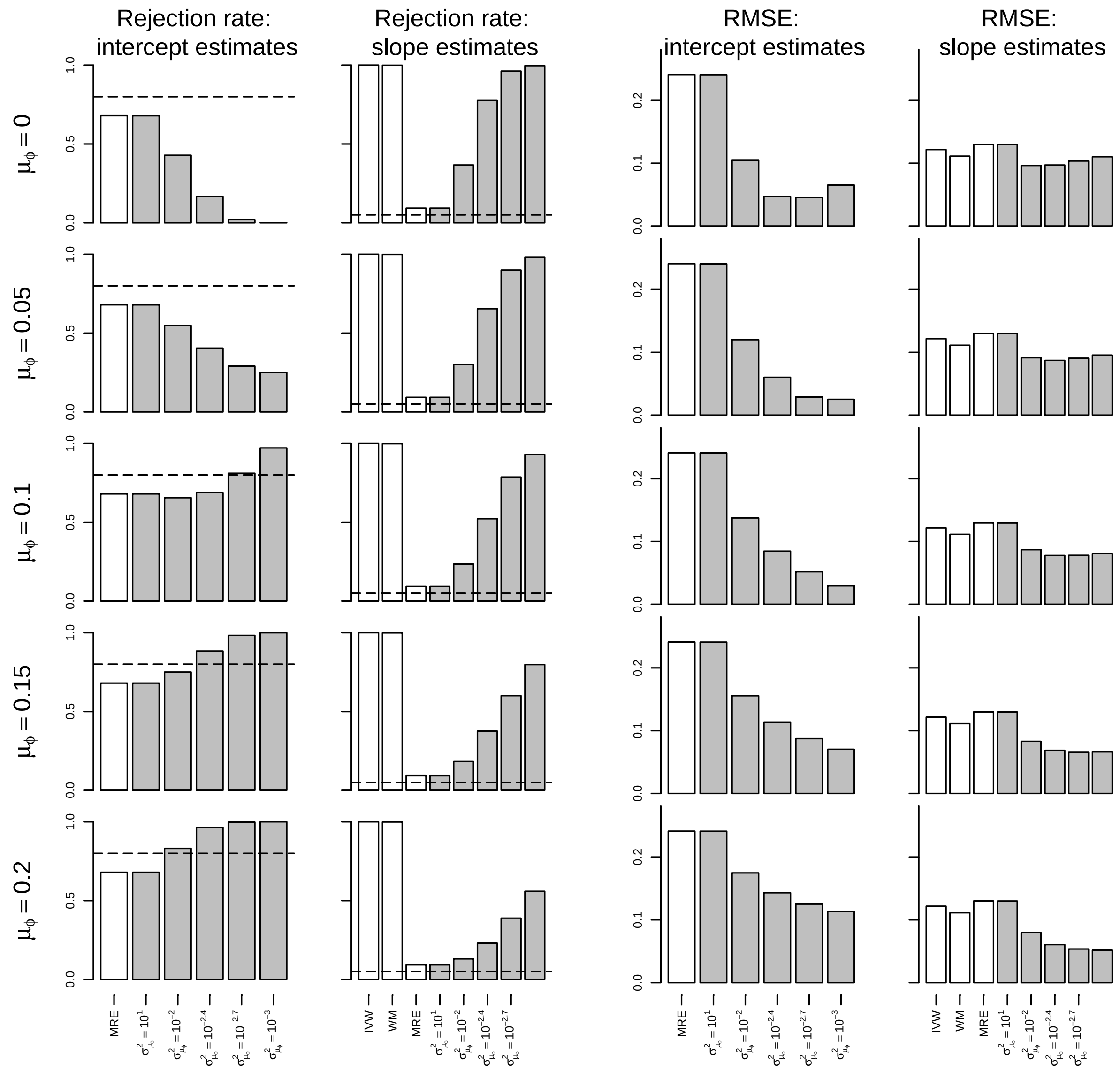

Figure 11: Rejection rate and root mean square error (RMSE) of a Mendelian randomization study using instrumental variable analyses with and without weakly informative Bayesian priors on the intercept (pleiotropy) term. Scenario III with pleiotropy, the InSIDE assumption violated and no effect of the phenotype on the outcome; $\phi_j = 0.10$ and $\theta = 0.00$, with J 20 independent SNPs, sampled from $n = 1,000$ subjects, and 5,000 replications. IVW = inverse variance weighted instrumental variable analysis without Egger correction; WM = Weighted Median estimator; MRE = Mendelian Randomization study using an instrumental variable analysis with Egger correction; μ_ϕ and $\sigma^2_{\mu_\phi}$ are the prior hyperparameters for the mean and variance of the Bayesian MRE.

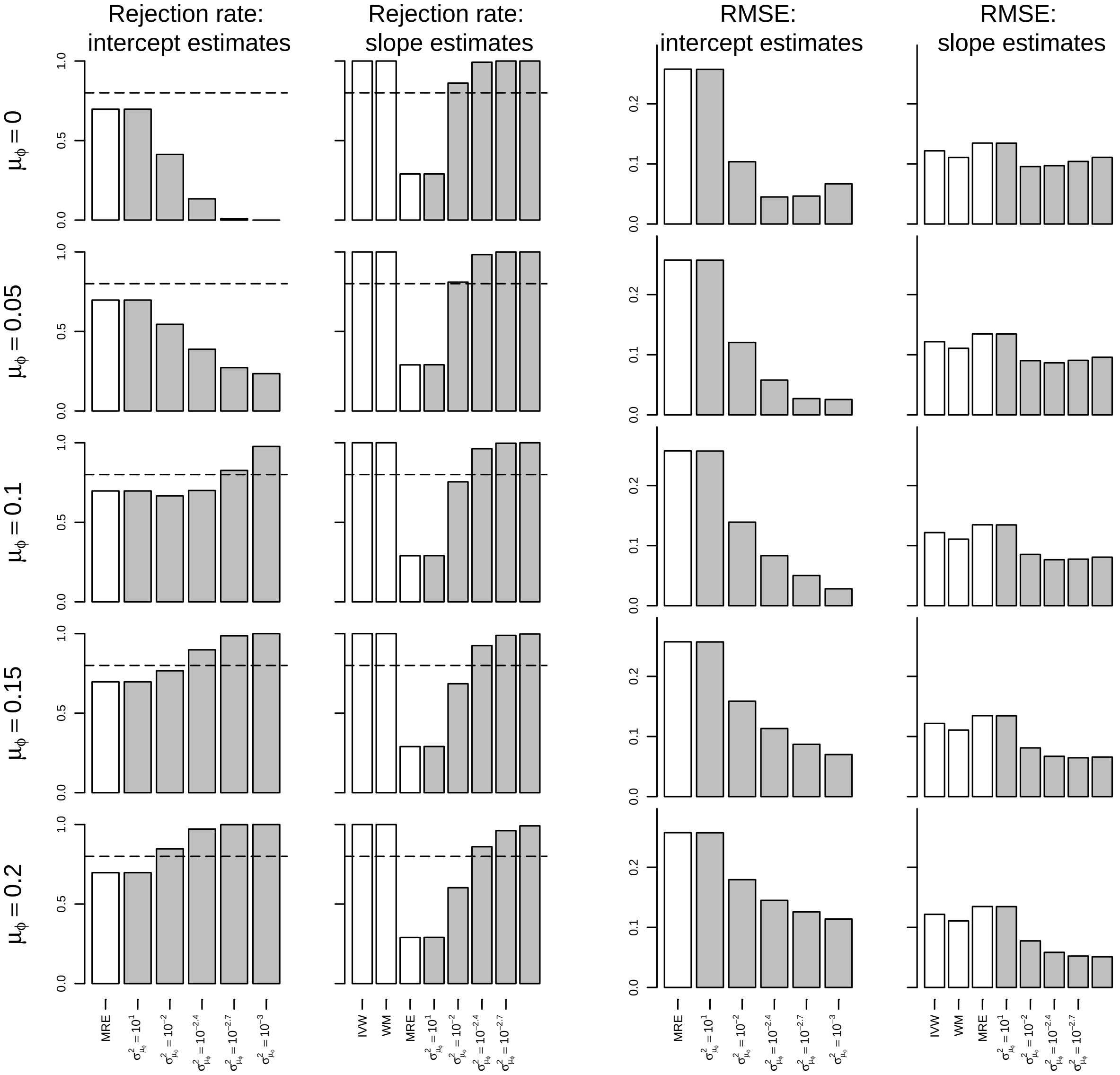

Figure 12: Rejection rate and root mean square error (RMSE) of a Mendelian randomization study using instrumental variable analyses with and without weakly informative Bayesian priors on the intercept (pleiotropy) term. Scenario III with pleiotropy, the InSIDE assumption violated and a causal effect of the phenotype on the outcome; $\phi_j = 0.10$ and $\theta = 0.05$, with J 20 independent SNPs, sampled from $n = 1,000$ subjects, and 5,000 replications. IVW = inverse variance weighted instrumental variable analysis without Egger correction; WM = Weighted Median estimator; MRE = Mendelian Randomization study using an instrumental variable analysis with Egger correction; μ_ϕ and $\sigma^2_{\mu_\phi}$ are the prior hyperparameters for the mean and variance of the Bayesian MRE.

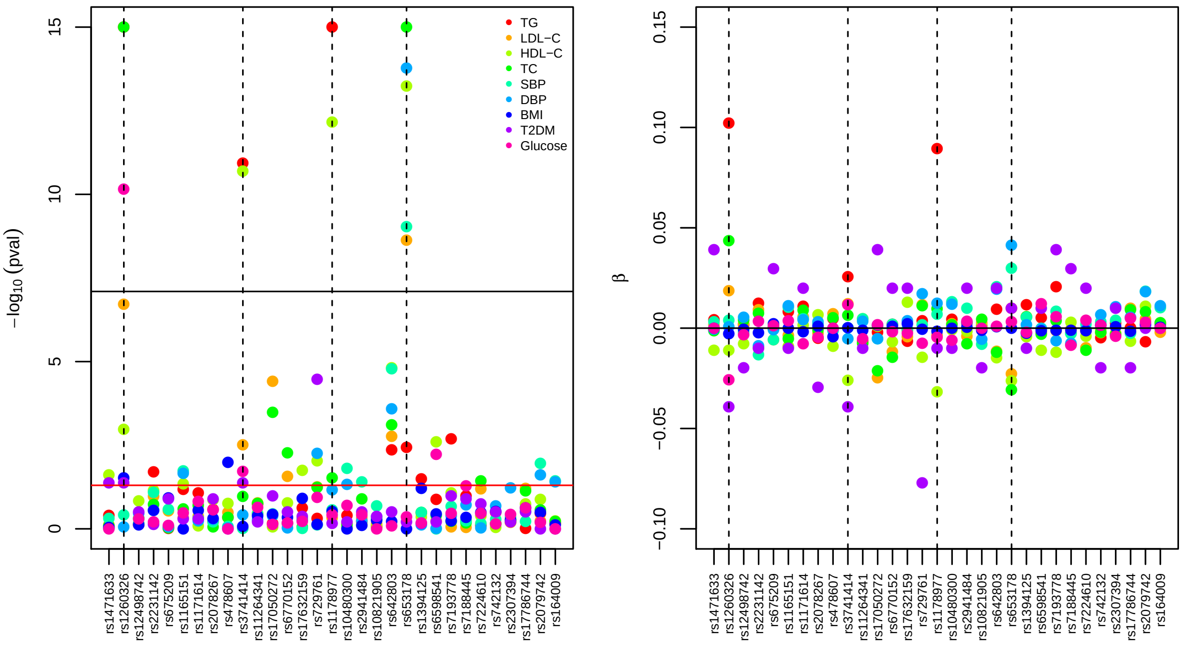

Figure 13: Observed pleiotropy for 31 SNPs related to plasma urate. Left panel $-log_{10}(p - values)$, with the horizontal black line indicating a genome wide significance alpha of $8 * 10^{-8}$ and the red line an alpha of 0.05. The right panel depicts slope coefficients, either representing a mean difference (per standard deviation) or $log\,(OR)$. Vertical lines indicate the SNPs with pleiotropic effects passing the genome-wide significance threshold.
